# Impact of personalized coaching on the use of digital health interventions for movement therapy in rheumatology: a randomized controlled trial

**DOI:** 10.1038/s41598-026-59770-7

**Published:** 2026-06-24

**Authors:** Magdalena Binder, Alp Temiz, Paloma Palm von Alten Blaskowitz, Birte Coppers, Ines Ebner, Pascal Petit, Nicolas Vuillerme, Sebastian Rudolf, Valerie Schlaht, Johannes Knitza, Anna-Maria Liphardt, Georg Schett, Harriet Morf

**Affiliations:** 1 Department of Internal Medicine 3- Rheumatology & Immunology, Universitätsklinikum Erlangen, Friedrich-Alexander-Universität Erlangen-Nürnberg, Ulmenweg 18, 91054 Erlangen, Germany; 2 Deutsches Zentrum Immuntherapie, Universitätsklinikum Erlangen, Friedrich-Alexander-Universität Erlangen-Nürnberg, Erlangen, Germany; 3 Univ. Grenoble Alpes, CNRS, Grenoble INP, LIG Sangria, Grenoble, France; 4 Institut Universitaire de France, Paris, France; 5 Institute for Digital Medicine, University Hospital of Giessen and Marburg, Philipps-University Marburg, Marburg, Germany

**Keywords:** Axial spondylarthritis, Spondyloarthritis, Digital health application, DHA, ViViRA, Kaia, Movement therapy, e-Health, Physical function, Mobility, Diseases, Health care, Medical research, Rheumatology

## Abstract

Spondyloarthropathies (SpA) are characterized by low back pain and limited mobility. Therefore, physical activity (PA) is an essential part of the treatment, yielding positive effects on clinical symptoms. Digital health applications (DHAs) present new opportunities to promote clinical outcomes, however, their long-term effectiveness is often limited by low adherence and high dropout rates.This study investigates whether integrating personalized or AI-driven coaching enhances the therapeutic benefits of DHA in patients with SpA. SpAs patients were randomized into one of 3 groups. They were instructed to exercise at least 2–3 times per week for 6 months with the DHA according to their group (intervention groups: ViViRA (with personal coaching) or Kaia Health (with AI-based coaching); control group: ViViRA (without coaching)). Personal coaching consisted of a one-time, 30-min online coaching session prior to using DHA, while the AI coaching consisted of video-based AI integrated into DHA to provide movement guidance during each session. At baseline, after 3 and 6 months sociodemographic, questionnaires and mobility were assessed. Data from 78 participants were analyzed (mean age 51 years; 68% female). All three digital interventions showed a significant improvement in mobility (Bath Ankylosing Spondylitis Metrology Index (BASM), range: 0–10, lower scores = better mobility; BL-3 month: mean BASMI change − 0.6 to − 0.7; all *p* < 0.001). Pain intensity decreased substantially in all arms (PainDETECT, neuropathic pain, range: 0–38, higher scores = more severe pain; BL-6 month: mean reduction − 4.6 to − 6.6 points; all *p* ≤ 0.006). PAHCO (Physical Activity-related Health Competence) control competence increased over time and reached statistical significance only in the ViViRA + coaching group (PAHCO: higher scores = better physical activity-related health competence; BL-6 month: + 1.02, *p* = 0.013) but did not exceed the other interventions in a direct comparison. Overall, none of the coaching strategies showed significant superiority over the stand-alone digital therapy. Adherence was the same in all groups after 3 months (2–3 weekly use of DHA). Digital movement therapy with the use of DHA improves mobility and pain independently of coaching in SpAs patients. In contrast, personal coaching has been shown to improve health-related skills which could indicate potential benefits for self-management and long-term treatment adherence.

*Trial registration* The study is registered in the German clinical trial registry (DRKS) under the following ID: DRKS00035191, https://www.drks.de/search/de/trial/DRKS00035191/details, Registration date: 01.10.2024.

**Supplementary Information:**

The online version contains supplementary material available at 10.1038/s41598-026-59770-7.

## Introduction

Spondyloarthropathies (SpAs) are a group of chronic inflammatory diseases characterized by chronic back pain and progressive stiffness of the spine, if untreated. Typically, it manifests in younger age and with a complex picture of different symptoms^1^. In addition, not only joints are affected, but also inflammation of the skin (psoriasis), intestines (chronic inflammatory bowel disease), or eyes (uveitis) can occur.

Treatment is based on pharmacological and non-pharmacological interventions to achieve the therapeutic goals of pain relief, preservation of physical function and work capacity and prevention of structural lesions^1^. Non-pharmacological interventions include physical activity (PA), which has overall positive effects on disease activity, physical function, and mobility^2^. PA is recommended by the European Alliance of Associations for Rheumatology (EULAR) as a standard component of SpA treatment to reduce disease symptoms and inflammatory activity ^3^. In addition, PA exerts broader beneficial effects on physical function and mobility, underlining its role as an important complementary therapy. Recent studies demonstrated that exercise programs that combine flexibility and/or endurance training and group training programs led to better results, especially in terms of mobility, compared to individual home programs. Patient education, active patient participation, and motivation are considered important factors influencing the effectiveness of PA interventions^4^. These factors can be promoted through targeted educational measures and the active involvement of patients in the therapy process^4^. A new way to motivate patients to exercise and give them easy access to PA are digital health applications (DHAs).

DHAs are diagnostically and therapeutically effective, low-risk CE-labelled digital medical devices. Applications can be app- or browser-based and can be prescribed and covered by the health insurance since December 2019 as part of the ‘Digital Healthcare Act’ (Digitale-Versorgung-Gesetz) in Germany^5^. For their listing and authorization, DHAs must demonstrate a medical benefit in their application as well as patient-relevant structural and procedural improvements, including in the areas of coordination of treatment processes, facilitation of access to care or health literacy^6^.

The use of domain-specific DHA in various clinical areas has already proven successful, for example in the treatment of diabetes mellitus or depression^7^. However, there are currently no DHAs in Germany that are specifically approved for rheumatology^5^. Nevertheless, there are some apps that have already been used and tested in studies with rheumatology patient groups as well as DHAs in development that are specifically designed for rheumatic diseases, such as the Axia app for SpA patients, which includes meditation, education and more than 250 exercise videos. The use of this app has also shown positive results in terms of pain and mobility^8^. According to a recent review from 2024, most commonly prescribed DHAs in rheumatology include Zanadio for weight reduction, ViViRA for back pain, and Kalmeda for tinnitus management^9^.

The demand for DHAs in rheumatology is high, especially in patients with SpA. A German study from 2021 showed that 84% of SpA patients (n = 435) saw a need for an app specifically designed for SpA^10^. Recently, a study investigating the use of movement-based digital applications in patients with SpA reported positive effects on pain, strength, and mobility outcomes^11^. Two observational studies (2023, 2024) on real-world application data for the DHA ViViRA in cases of non-specific back pain showed a significant and clinically relevant reduction in back pain after 12 weeks of use, as well as an improvement in functional scores. The positive effect was particularly evident after just 8 weeks. However, both studies also recorded high dropout rates, which limits the interpretation of the results^11,12^.

Nevertheless, a 2023 review with 29 trials identified various system-related challenges in the use of DHAs, including low adherence and high drop-out rates across a broad range of clinical contexts^13^. Although positive results and a high level of patient acceptance when using DHAs can be shown, the drop-out rates in the studies and long-term non-compliance in app use are high^14^. According to a systematic review of digital self-help interventions for common mental health problems, evidence of full or sustained use ranged from 0.5 to 28.6%^15^.

One approach to increase long-term adherence in the use of the apps is to combine their use with coaching or personal support. According to Glanz et al., coaching is a concept based on several behavioral models and theories that aims to promote health-enhancing behavioral changes through individual support, joint goal-setting, ongoing guidance, and regular feedback^16^. A meta-analysis from 2020, for example, examined the effects of health coaching on behavior change in adults with cardiovascular risk factors and reported a small but significant positive effect on increasing PA, dietary behavior, health behavior, and stress management. Coaching involved a combination of goal setting, motivational interviewing, and interdisciplinary teamwork^17^. There is also evidence that digital assistance systems, such as wearable sensors, intelligent software or personalized feedback, can also be used in coaching for chronic illnesses^18^.

The aim of this study is to evaluate the impact of personalized coaching and artificial intelligence (AI) coaching on the benefits of clinical outcomes, adherence and PA when using DHAs in patients with SpA.

## Methods

### Study design and patient recruitment

This monocentric, prospective, randomized, controlled study was conducted between March 2024 and June 2025. Participants for the intervention and control groups were recruited from the outpatient clinics of the Department of Rheumatology and Immunology at the Universitätsklinikum Erlangen, Germany. 78 patients with SpA^19^ over 18 years of age were included*.* The enrollment and follow-up of study participants were presented using a CONSORT 2025 flow diagram (Supplement S1). General exclusion criteria include pregnancy, changes in immunosuppressive therapy in the last three months before inclusion in the study, prior use of ViViRA or Kaia apps, participation in movement-related DHA studies, or previous movement coaching. App-specific exclusions involved malignant or secondary neoplasms and severe joint or spine conditions, including implant-related complications^20^ (Supplement S2).

### Ethical considerations

The study protocol was approved by the medical faculty ethics committee (Nr.: 24–86-B, 14.05.2024) of the Friedrich-Alexander-Universität Erlangen-Nürnberg, Erlangen, Germany and registered in the German Clinical Trials Register (DRKS-ID DRKS00035191, registration date: 01.10.2024). Participation in the study was voluntary. All patients provided their written informed consent before study inclusion. The participants were coded with a pseudonym. The collected data were stored and analyzed in a password-protected database REDCap (Research Electronic Data Capture)^21^, and only previously defined and authorized persons had access. Patients had the option of withdrawing their participation in the study at any time, whereby all personal data were irrevocably deleted. The study was conducted in accordance with the ethical guidelines of the Declaration of Helsinki.

### Measurements

After enrollment and assignment of a unique study number, patients were assigned to one of the three study groups using a computer-generated randomization sequence (1:1:1) generated by our statistician: the ViViRA intervention group with personal coaching (Intervention Coaching (IC) ViViRA), the Kaia intervention group with AI coaching (IC Kaia), or the ViViRA control group (Control Group (CG) ViViRA. Subsequently, the patients answered questionnaires and baseline measurements were taken. The questionnaires were collected digitally via REDCap. Sociodemographic data were collected at baseline. Spinal mobility was assessed with the Bath Ankylosing Spondylitis Metrology Index (BASMI) [0–10, where 0 is not limited and 10 highly limited] (measured by tape)^22^. After three months (Follow- Up (FU)1), both the questionnaires and the measurements (BASMI) were repeated, followed by the collection of questionnaires again after six (FU2), nine (FU3) and 12 (FU4) months. In addition to the quantitative analyses, exploratory qualitative feedback was collected via email after six months. Participants who agreed responded once in writing; no follow-up questions were conducted.

### Patient reported outcome measures (PROMs)

Because DHAs and coaching can influence both physical and behavioral aspects of exercise therapy, clinical, functional, and health-related PROMs were collected. To assess clinical and functional symptoms of SpA, specific disease related questionnaires for functional disability (BASFI = Bath Ankylosing Spondylitis Functional Index; range 0–10, with higher scores indicating greater disability), physical limitations (HAQ = Health Assessment Questionnaire; 0–3, with higher scores indicating greater impairment), and disease activity (BASDAI = Bath Ankylosing Spondylitis Disease Activity Index; 0–10, with higher values indicating greater disease activity) were assessed. Pain was assessed using a visual analog scale (VAS; 0–10, with higher values indicating greater pain intensity) and the painDETECT questionnaire (0–38, with higher values indicating stronger neuropathic pain characteristics). In addition, behavioral outcomes related to PA (IPAQ = International Physical Activity Questionnaire; MET-minutes/week, with higher values indicating higher PA), fear of movement (TSK = Tampa Scale of Kinesiophobia; 17–68, with higher scores indicating greater fear of exercise) and PA-related health competence (PAHCO = Physical Activity-related Health Competence; multidimensional Likert scale (1–5), with higher scores indicating better health competence) were recorded to examine potential coaching-related effects on self-management and exercise behavior. Theses questionnaires are used as standard PROMs in movement therapy trials. Fatigue (FACIT-F = Functional Assessment of Chronic Illness Therapy – Fatigue; 0–52, with higher scores indicating less fatigue), sleep quality (PSQI = Pittsburgh Sleep Quality Index; 0–21, with higher scores indicating more sleep disturbance), and quality of life (SF-36 = Short Form Health Survey (36 items); 0–100, with higher scores indicating better quality of life) were included to capture the broader health-related effects of the intervention (Table 1). Furthermore, compliance and engagement were assessed qualitatively, for example with questions in questionnaires such as “Do you still use the app?” or “What was your main motivation for training?” (Supplement S3). Instruments used in this study have been previously validated and are well established as reliable tools in both clinical and digital health research (see references in Table 1).

**Table 1:** Questionnaires used in the study, conducted from June 2024 until July 2025 at the outpatient clinics of the Department of Rheumatology and Immunology at the university hospital Erlangen, Germany.

Questionnaires	Range	Measurement	References
**BASDAI** [score] *Bath Ankylosing Spondylitis Disease Activity Index*	0 to 10 (cut off = 4): 0 = no limitations, 10 = strongest possible limitations < *4 low disease activity* ≥ *4 severe disease activity*	Measurement of disease activity in SpA	^23^
**BASFI** [score] *Bath Ankylosing Spondylitis Functional Index*	0 to 10: 0 = no limitations, 10 = strongest possible limitations	Measurement of functional limitation in ankylosing spondylitis	^23^
**Pain Detect** [score]	0 to 38: 0–12 = neuropathic pain component unlikely, 13–18 = uncertain or possible 19–38 = neuropathic pain component probably	Measurement of neuropathic pain	^24^
**TSK [scale]** *Tampa Scale of Kinesiophobia*	17 to 68: < 37 = low degree of kinesiophobia, ≥ 37 = high degree of kinesiophobia	Fear of movement caused by pain or fear of pain	^25^
**PAHCO [scale]** *Physical Activity-related Health Competence*	Multidimensional Likert scales (e.g., 1–5) Higher values = better competence	PA related skills: movement competence, control competence, self-regulation competence	^26^
**HAQ** [score] *Health Assessment Questionnaire*	0 = no difficulties 0–1 = no to mild difficulties, 1–2 = mild to major difficulties, 2–3 = severe to very severe difficulties	Physical impairment in rheumatic diseases	^23^
**Facit-F** [score] *Functional Assessment of Chronic Illness Therapy – Fatigue*	0 to 52: Higher score indicates less fatigue	Measurement of Fatigue in patients with chronic diseases	^27^
**SF-36** [score] *Short Form Health survey (36 items)*	0 to 100: higher score indicates a better health status	Assessment of Health-related quality of life [physical component score (PCS) and mental component score (MCS)]	^28^
**PSQI** [score] *Pittsburgh Sleep Quality Index*	0 to 21: > 5 indicates clinically relevant sleep disorder	Assessment of the subjective sleep quality in the past 4 weeks	^29^
**IPAQ** [score] *International Physical Activity Score*	MET-minutes/week (metabolic equivalent of task), < 600 = movement below the minimum level 600–3000 = meets the WHO recommendations > 3000 = high PA	Assessment of daily PA in the past 7 days	^30^

### Digital health applications

A DHA was prescribed to every included patient for the first time on the day of baseline data collection. After prescription, patients could download the DHA with a code for use on a smartphone or tablet. It was necessary to sign an additional data protection declaration to use the DHA^20^. Following the initial prescription, patients were allowed to use the DHA for a 3-month period. At the follow-up visit, a new prescription was issued for the subsequent 3 months. Referring to their randomly assigned group, the patients either used the Kaia Health application (IC Kaia) or the ViViRA application (IC ViViRA and CG ViViRA).

IC/CG ViViRA: Patients using the ViViRA application were required to complete 4 exercises per unit daily, with a maximum time commitment of 15 min. These exercises were also based on repetitions or time and could be adjusted for difficulty. After each exercise, patients were requested to provide feedback on their exercise execution, and the exercise protocol (intensity and complexity) was consequently adapted automatically by the DHA according to the individual fitness and pain level. Performance during the training sessions and the evolution of pain and mobility are visualized in the activity history of the DHA.

IC Kaia: Patients using the DHA Kaia were instructed to perform daily 3–5 exercises per unit lasting between 10 and 30 min. These exercises consisted of short fitness activities, either based on repetitions or time (e.g., squats or plank holds). The training duration and intensity can be adjusted during training. After training, patients should provide feedback on the level of difficulty of the exercises so that the training can be customized. The AI-driven coaching of the DHA Kaia analyzes posture during each exercise and provides real-time corrective feedback via webcam.

### Intervention

IC ViViRA + personal coaching: Patients using the ViViRA application scheduled an individual 30-min online coaching session via Zoom at the beginning of the study after completing the baseline questionnaires before using the application the first time, which included practical assistance with physical exercises as well as personalized advice and goal setting. Issues and challenges were discussed, and personal goals were established. The goal was to strengthen adherence to the app usage. A standardized protocol was used, which, however, focused individually on personal hurdles and goals (Supplement S4).

IC Kaia + AI coaching: The Kaia Motion Coach is an AI-powered application that analyzes patients’ movements in real time using the smartphone’s selfie camera. Through digital markers and immediate feedback, it highlights areas for improvement in posture and movement execution. The Motion Coach helps enhance training quality, reduce the risk of injury, and enables patients to exercise independently, anytime and anywhere^31^.

### Control

CG ViViRA without personal coaching: The participants in the control group were prescribed the ViViRA application for 24 weeks without personal or AI-powered Coaching. The key characteristics and differences between study arms are summarized in Table 2.

**Table 2:** Overview of studies arms according to FITT (Frequency, Intensity, Time, Type) principle.

Group	DHA	Frequency	Time per session	Type of intervention	Intensity/adaptation	Additional component
IC Kaia	Kaia health	at least 2–3 times a week	10-30 min	4 structured exercises per session (repetitions or time-based)	App-based adaptive adjustment based on pain and fitness feedback	Motion Coach (AI movement analysis and real-time posture feedback via camera)
IC ViViRA	ViViRA		≤ 15 min	3–5 functional exercises (repetitions or time-based)	App-based adaptive adjustment based on pain and fitness feedback	30-min standardized online coaching session at baseline
CG ViViRA						–

### Statistical analysis

Baseline characteristics were summarized as mean (Standard Deviation (SD) for continuous variables and counts (percentages) for categorical variables. For each predefined outcome, a linear mixed-effects model was fitted with fixed effects for visit, intervention group, and their interaction, and a random intercept for participants to account for repeated measurements. Estimated marginal means and pairwise comparisons were derived from the fitted models using model-based standard errors. Group differences at each visit, as well as within-group changes across visits, were quantified using estimated marginal mean contrasts. Results are reported with confidence intervals and corresponding p-values. Visualizations and structured output tables were generated directly from the model estimates. All analyses were conducted in R version 4.5.1.

## Results

The study included 78 patients with SpA from the University Hospital Erlangen-Nuremberg. Data from n = 25 in IC ViViRA, n = 27 in the IC Kaia and n = 26 in the CG ViViRA were evaluated. At baseline, the groups were balanced in terms of potential influencing factors. Descriptive baseline characteristics of the included participants are presented in Supplement S5 and S6. There were no indications of systematic differences in demographics (age, gender, body mass index (BMI)), disease severity (duration of disease, BASDAI, BASMI), functional values (BASFI, HAQ), or medication (all *p* > 0.05). This allows for valid intergroup comparability in the adjusted and unadjusted analyses. There were no significant differences between the three intervention groups (all *p* ≥ 0.05).

### Mobility

**Fig. 1:**
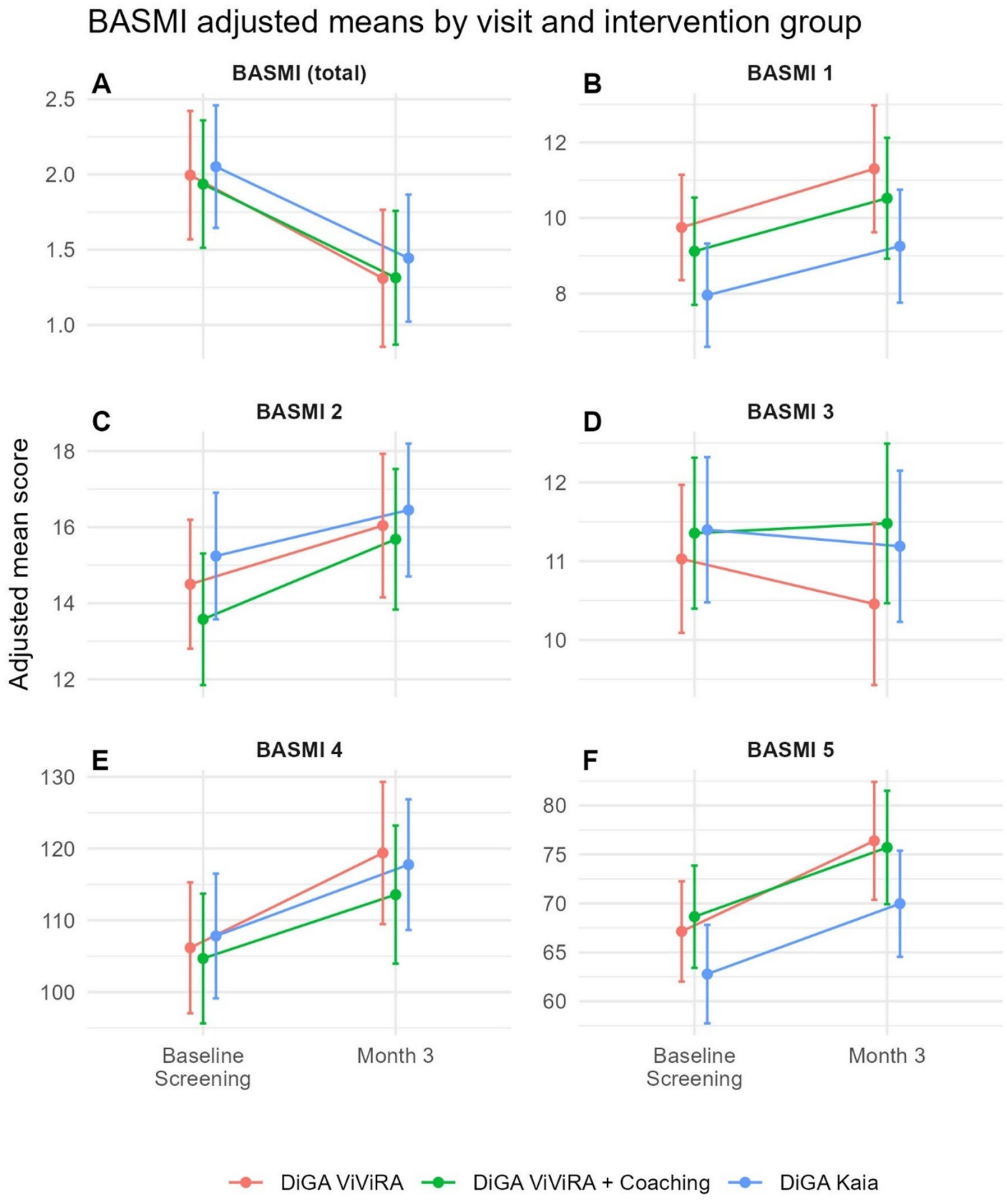
Adjusted BASMI total and component scores by visit and intervention group (DiGA ViViRA = CG ViViRA, DiGA ViViRA + Coaching = IC ViViRA, DiGA Kaia = IC Kaia).

Spinal mobility was assessed using BASMI (range 0–10; higher scores indicate poorer mobility). All three groups showed a significant improvement in the score after 3 months (FU1) (average − 0.6 to − 0.7 points, p < 0.001 in all arms). There were no significant differences between the groups at any point in time (see Fig. 1). The results of the BASMI subgroups confirm the overall result (see Fig. 1).

Estimated marginal means of the Bath Ankylosing Spondylitis Metrology Index (BASMI) total score (panel A) and individual BASMI components (panels B–F) at baseline and month 3, stratified by intervention group. BASMI was not assessed at month 6. Points represent adjusted means and error bars indicate 95% confidence intervals derived from the fitted models. BASMI total (panel A) is shown on the standardized BASMI linear scale, whereas BASMI components (panels B–F) are displayed on their original measurement scales. Lower BASMI total scores indicate better overall spinal mobility. For the individual BASMI components, lower scores indicate improvement for tragus-to-wall distance (BASMI 1), whereas higher scores indicate improvement for lumbar side flexion (BASMI 2), cervical rotation (BASMI 3), modified Schober test (BASMI 4), and intermalleolar distance (BASMI 5).

### Pain

**Fig. 2:**
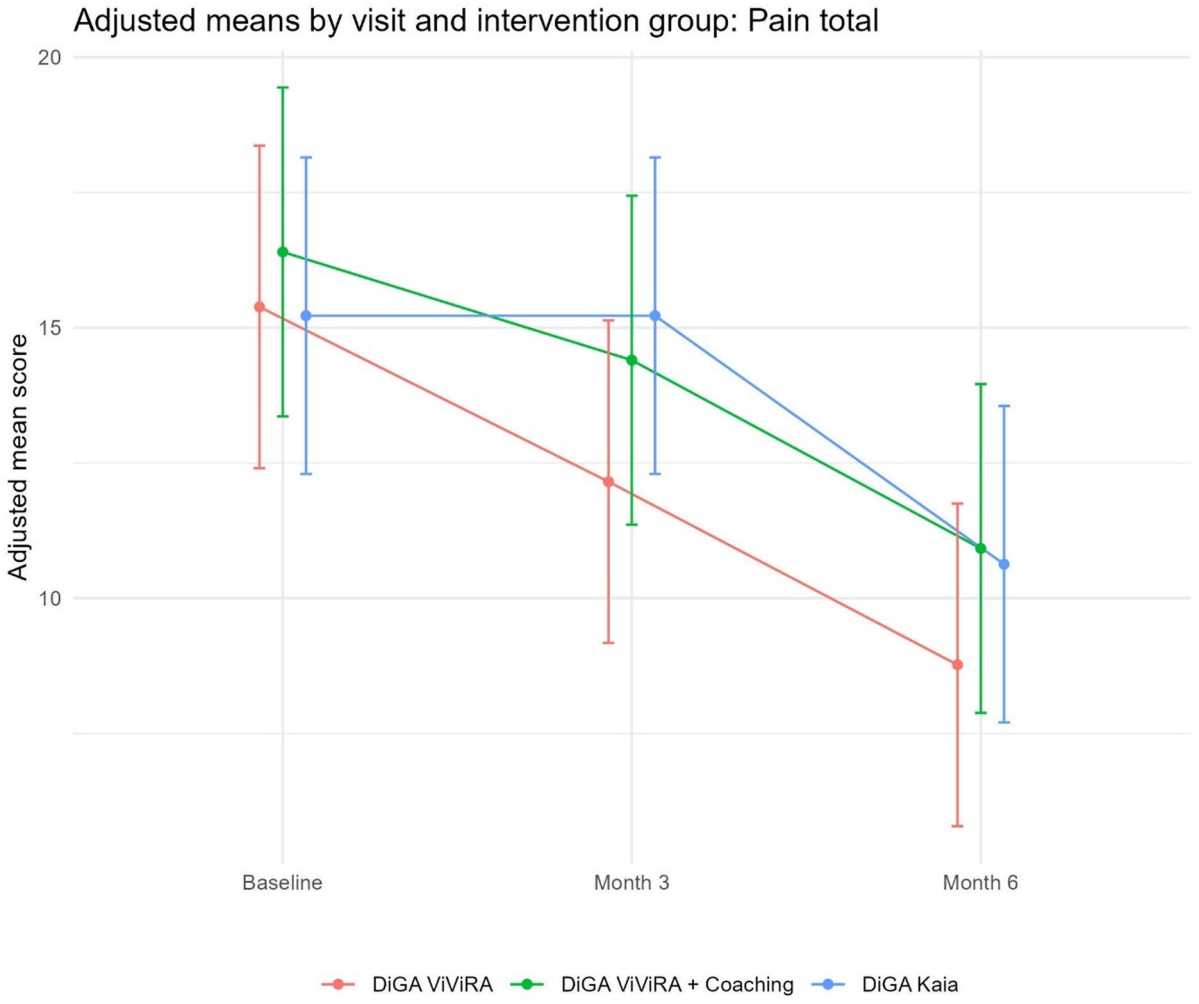
Adjusted pain total scores by visit and intervention group (DiGA ViViRA = CG ViViRA, DiGA ViViRA + Coaching = IC ViViRA, DiGA Kaia = IC Kaia). Estimated marginal means of pain total at baseline, month 3, and month 6 by intervention group. Points indicate adjusted means and error bars show 95% confidence intervals. Lower scores indicate less pain and therefore reflect improvement in pain symptoms.

The painDETECT questionnaire, ranges from 0 to 38, with higher scores indicating more severe neuropathic pain symptoms, showed a significant reduction in neuropathic pain intensity in all groups up to month 6 compared to baseline (IC Kaia: − 4.6, *p* ≤ 0.006; IC ViViRA: − 5.5, *p* ≤ 0.001; CG ViViRA − 6.6 points, *p* ≤ 0.006). The values thus improved in all groups from an average of about 15–16 across arms (baseline) to mean values of about 8.8–10.9 in all arms (see Fig. 2). There were no significant differences between the groups.

### Physical activity-related health competence

**Fig. 3:**
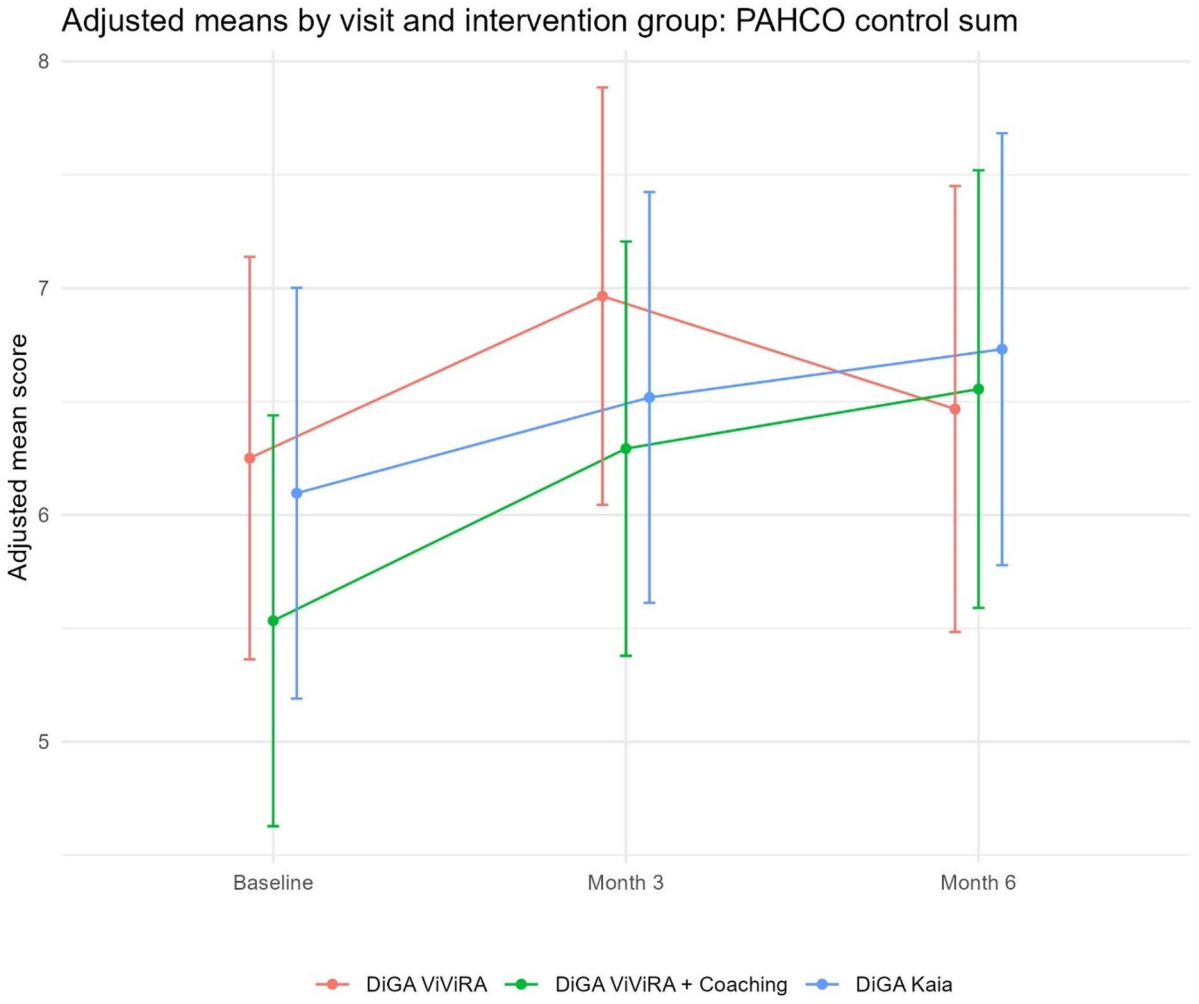
Adjusted PAHCO control sum scores by visit and intervention group (DiGA ViViRA = CG ViViRA, DiGA ViViRA + Coaching = IC ViViRA, DiGA Kaia = IC Kaia). Estimated marginal means of PAHCO control sum at baseline, month 3, and month 6 by intervention group. Points indicate adjusted means and error bars show 95% confidence intervals. Higher scores indicate greater control competence and therefore reflect improvement in this domain.

**Fig. 4:**
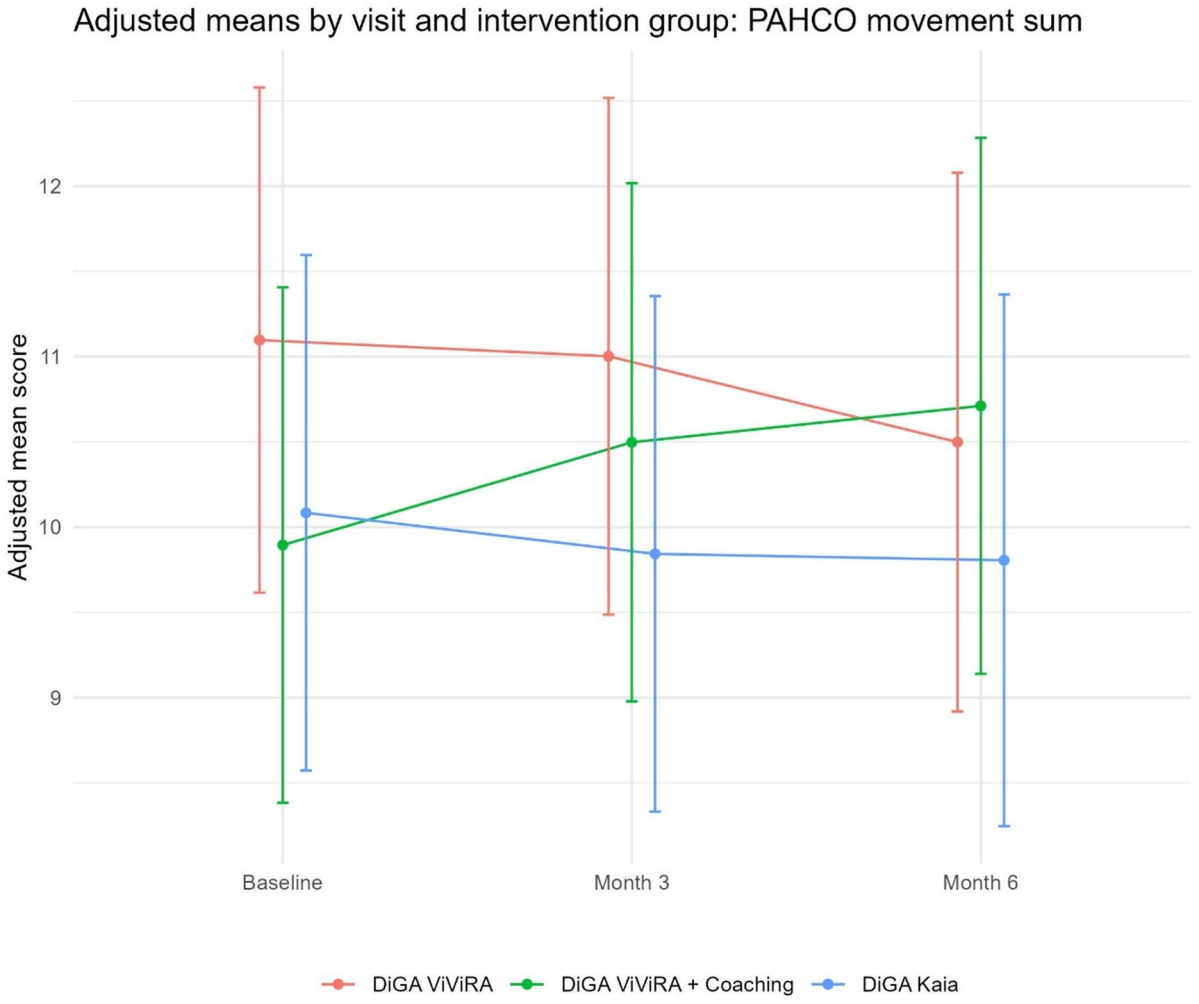
Adjusted PAHCO movement sum scores by visit and intervention group (DiGA ViViRA = CG ViViRA, DiGA ViViRA + Coaching = IC ViViRA, DiGA Kaia = IC Kaia). Estimated marginal means of PAHCO movementsum at baseline, month 3, and month 6 by intervention group. Points indicate adjusted means and error bars show 95% confidence intervals. Higher scores indicate greater movement competence and therefore reflect improvement in this domain.

**Fig. 5:**
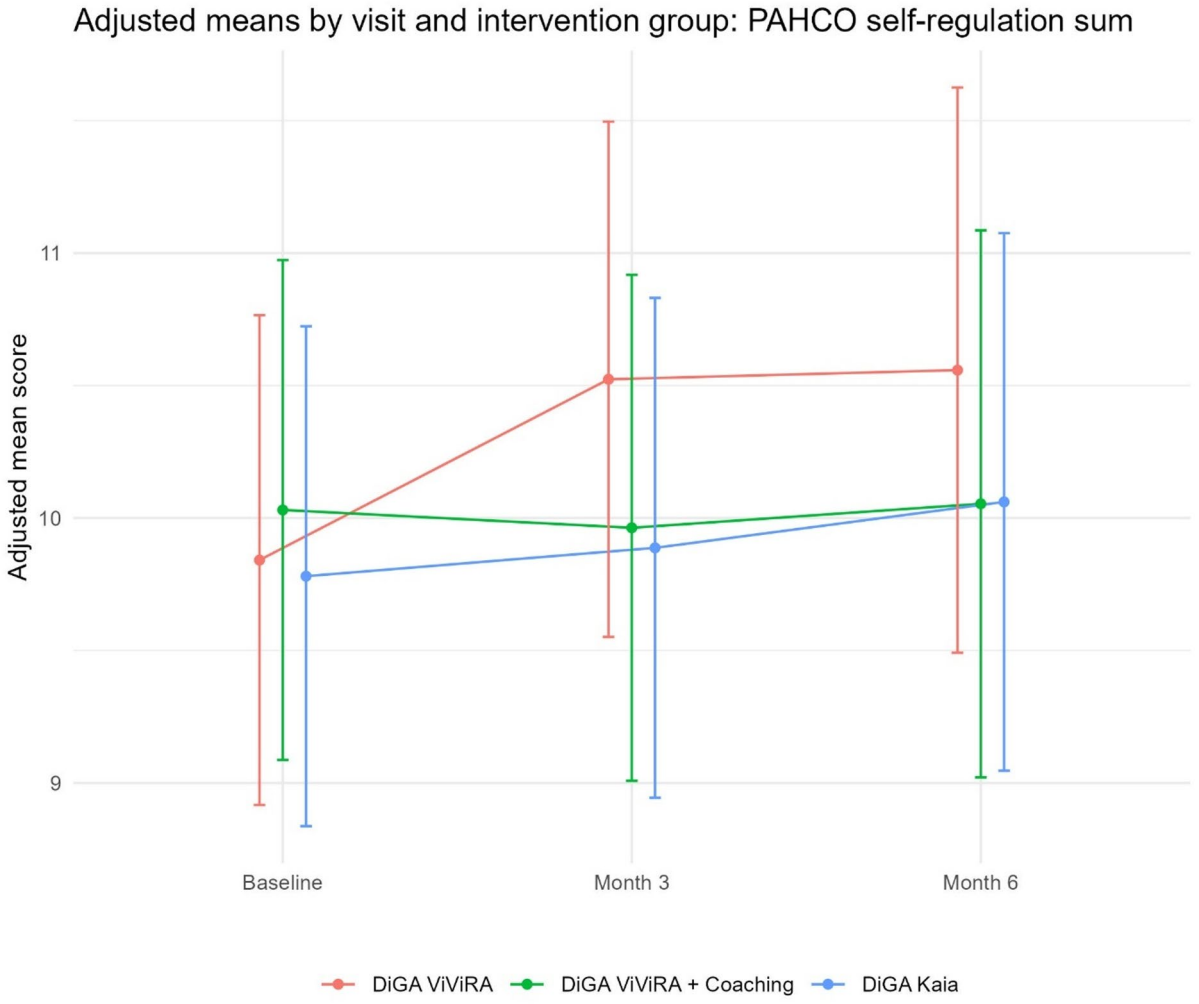
Adjusted PAHCO self-regulation sum scores by visit and intervention group (DiGA ViViRA = CG ViViRA, DiGA ViViRA + Coaching = IC ViViRA, DiGA Kaia = IC Kaia). Estimated marginal means of PAHCO self-regulationsum at baseline, month 3, and month 6 by intervention group. Points indicate adjusted meansand error bars show 95% confidence intervals. Higher scores indicate greater self-regulation competence and therefore reflect improvement in this domain.

The total control competence score in the PAHCO questionnaire (higher scores = better physical activity-related health competence) increased in all groups over the course of the study, but most in the IC ViViRA, where a significant change was observed (baseline − FU1 ≈ + 0.76, *p* = 0.046; baseline − FU2 ≈ + 1.02, *p* = 0.013). Similar trends of improvement were observed in the IC Kaia and CG ViViRA groups, but these were not significant (*p* ≥ 0.08) (see Figs. 3, 4, 5).

### Functionality and disease activity

Overall, only minor changes were observed across groups for functionality and disease activity measure. BASFI (range 0–10, higher scores = more functional limitations ) showed a small, borderline significant improvement in the IG ViViRA at 3 and 6 months, while remaining stable or slightly reduced in the CG ViViRA and IG Kaia. BASDAI and HAQ showed no relevant changes in any group over the 6-month period.

### Quality of life

**Fig. 6:**
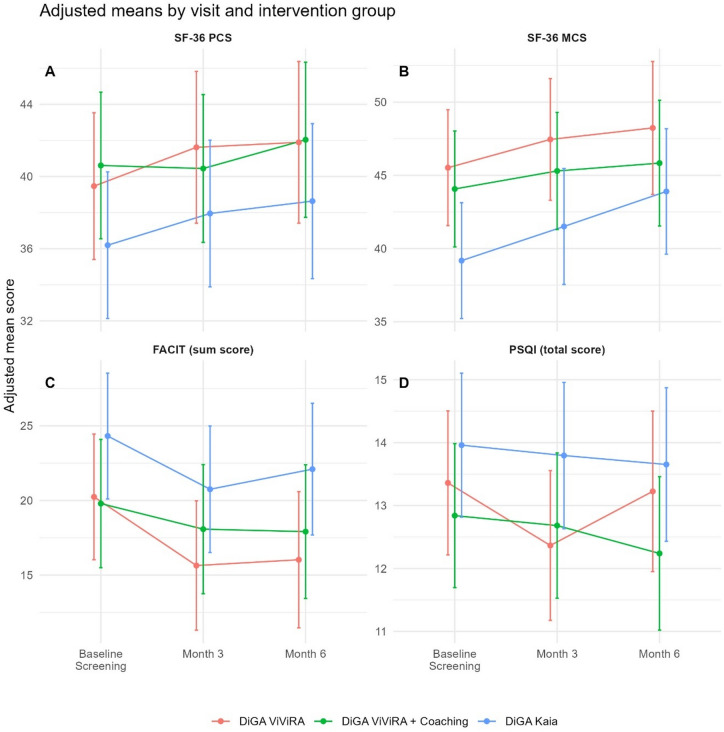
Adjusted quality of life, fatigue, and sleep scores by visit and intervention group (DiGA ViViRA = CG ViViRA, DiGA ViViRA + Coaching = IC ViViRA, DiGA Kaia = IC Kaia). Estimated marginal means by intervention group at baseline, month 3, and month 6 for (**A**) SF-36 PCS, (**B**) SF-36 MCS, (**C**) FACIT fatigue sum score, and (**D**) PSQI total score. Points indicate adjusted means and error bars show 95% confidence intervals. SF-36 PCS and MCS scores range from 0 to 100, with higher scores indicating better health-related quality of life. FACIT scores range from 0 to 52, with higher scores indicating less fatigue and therefore reflecting improvement. PSQI scores range from 0 to 21, with lower scores indicating better sleep quality and therefore reflecting improvement.

Quality of life (SF-36: range 0–100: higher score = better health status) was comparable across the three study arms at baseline (see Fig. 6). Physical health, assessed by the SF 36 physical component summary (PCS) was similar between groups (see Fig. 6A), with mean scores of 39 (SD 12) in the CG ViViRA, 41 (SD 11) in the IC ViViRA, and 36 (SD 9) in the IC Kaia, without between group differences (*p* = 0.2).

Mental health, measured by the SF 36 mental component summary (MCS) showed a comparable pattern across arms (see Fig. 6B). Slightly lower values were observed in the IC Kaia, but this difference did not reach conventional levels of evidence (*p* = 0.081).

Fatigue related quality of life, assessed using the FACIT fatigue sum score (range 0–52: higher score = less fatigue), was broadly similar across groups, with mean values ranging from 20 to 24 and no meaningful between group differences (see Fig. 6C; *p* = 0.2). Sleep related quality of life, evaluated by the PSQI total score (range 0–21:

 > 5 indicates clinically relevant sleep disorder), did not differ substantially between intervention arms (see Fig. 6D). Mean sleep related measures and overall PSQI scores were comparable across groups (all *p* ≥ 0.3).

### Compliance

The self-reported training adherence of participants in the intervention groups is shown in the Supplements (Supplement S7). Adherence to DHA use was comparable across all groups during the first 3 months, with reported usage of approximately 2–3 sessions per week. Across both coached intervention arms, the perceived need for external supervision during exercise decreased over time, while most participants reported positive effects on mobility and pain. Perceived mobility improvements diminished over follow-up, whereas perceived pain relief increased. Participants in the Kaia group more frequently reported willingness to continue app use beyond the study period, particularly noting early improvements in mobility.

In addition to the quantitative analyses, exploratory patient interviews were conducted to contextualize the study findings. These interviews did not reveal distinct patient subgroups or identifiable influencing factors.

## Discussion

Our purpose was the evaluation of the impact of personalized and AI Coaching when using DHAs in movement therapy in SpA patients. The study shows that DHAs can lead to clinically significant improvements in mobility and pain perception when used regularly, up to three times weekly. BASMI scores improved by approximately 0.6–0.7 points after three months, indicating better spinal mobility, while painDETECT scores decreased from approximately − 4.6 to − 6.6 points at month 6, reflecting a reduction in neuropathic pain symptoms. These findings suggest clinically relevant improvements in two key domains of spondyloarthritis management. Improvements in these domains occurred in all groups, regardless of whether additional coaching was offered. DHAs for movement therapy therefore specifically address the main symptoms of the disease—pain and limited mobility—as defined as key treatment goals in the German guidelines, for example, emphasizing their clinical relevance^1^. The observed positive effect of DHAs in our population can be plausibly explained by the wide range of different movement units, active rather than passive mobilization compared to conventional physiotherapy, and the standardization of the exercise modules^11^. These findings are consistent with earlier studies reporting positive effects of DHAs on pain and mobility, including studies on ViViRA^32^, and Kaia^33^, as well as studies demonstrating improvements in mobility parameters^11,14^. Exploratory patient interviews generally confirmed the quantitative findings, with participants reporting that they had noticed improvements in their mobility and pain levels whilst using the DHA. However, no clear patient groups or influencing factors could be identified.

Pain and mobility improved in all three groups after three months. While personal coaching offered only minor or unclear additional benefits for these modalities, it did lead to a significantly greater improvement in health-related control competence. Therefore the PAHCO questionnaire was used and refers to a person’s ability to control PA in a way that is beneficial to their health and tailored to their individual needs^34^. The demonstrated effect of coaching is consistent with studies on hypertension^35^ or diabetes mellitus^36^ that show that coaching and similar interventions can specifically promote behavioral skills. The observed improvement in health-related control competence indicates a potentially sustainable benefit and contrasts with many studies that report predominantly short-term effects of interventions in the context of chronic diseases. Previous works show that positive effects often fade after the end of the intervention, especially when supportive conditions are no longer in place^37,38^. The assumption of a lasting effect is supported by the results of the PAHCO sample study^39^. It shows that PAHCO values, including control competence, remain largely stable over time. It suggests that PAHCO is a stable, developable competence. Given this context, the increase in control competence observed in the coaching group appears particularly significant. Additional a reduction of fear of motion is seen in this group that supports the benefit of movement coaching before starting a movement therapy. The movement coaching carried out at the beginning of the intervention (IC ViViRA), aimed to improve understanding of the disease and to encourage self-management, and self-efficacy. Since people with chronic diseases depend on stable health and control competence in order to manage their disease independently in the long term, the stable PAHCO values over time underscore the potentially sustainable benefits of the intervention. Increased control competence is central to the independent management of everyday life and therapy, as difficulties in dealing with health-related information are associated with poorer self-management and limited participation^40^. It is noteworthy that participants in the IC ViViRA showed this significant increase in control competence despite their persistently low motor competence. A similar pattern was observed in the IC Kaia. This pattern suggests that individuals with lower PA competence may particularly benefit from interventions that target cognitive-regulatory aspects of PA. One possible explanation is a compensation mechanism whereby individuals with lower motor skills rely more heavily on control skills to regulate and adapt PA in a health-oriented manner. Within the PAHCO framework, this underscores the independence of the PAHCO sub-skills and highlights the relevance of control skills for individuals with limited physical performance. The fact that similar but statistically insignificant trends were observed in the comparison groups with AI-based (exercise) coaching and the control group could indicate that personal coaching strengthens skills that go beyond purely physical aspects. At the same time, the lack of significant differences between the groups could be due to the fact that the apps used by all patients (ViViRA and Kaia) already have integrated skills-building tools that are available to patients for additional voluntary use for example breathing and relaxation techniques for stress reduction or knowledge units on symptoms^20,31^.

A key success factor in exercise therapy for SpA is adherence to therapy, especially in the context of self-management using DHAs^41^. There were positive indications of good adherence across all study groups (Supplement S6). After both three and six months, the majority of participants stated that they wanted to continue using the respective application beyond the study period or have it prescribed again. This indicates a generally high level of acceptance of the digital interventions. There were dropouts during the course of the study, with the highest number in the control group. Regardless of this, the overall dropout rate of 27 out of 78 participants was moderate for a DHA intervention study and showed a tendency toward better adherence in the intervention groups^42^. The literature describes a lack of personal support as one of the most common barriers to adherence in home- and app-based exercise therapy in SpA patients^41^. However, this obstacle was not reported by the majority of patients in the present study. The vast majority of participants stated that they did not need additional personal support, suggesting that the applications examined provided sufficient support in the context of a time-limited study framework.

There were several limitations to this study. Further studies with longer observation periods are needed to better analyze long-term effects^43^. Coaching effects may only become apparent after prolonged use, whereas many DHA studies only cover short follow-up periods. With approximately 25 people per arm, the study is relatively small, which may have prevented moderate effects from being detected. Increasing the number of participants would make it easier to detect and compare influencing factors and other effects.^44^. Coaching effects can be enhanced through repeated contact^45–47^. In this study, personal coaching took place only once; after that, participants trained independently with the app. More frequent coaching could increase the effect, although medical supervision is provided anyway when using DHAs^47^. The prescription and activation process was perceived as an obstacle, as the regular renewal and activation of the app disrupts the rhythm of use. Optimizing these processes could increase adherence and effectiveness^13^. Since the actual use of the applications was not objectively monitored in the present study, the assessment of adherence is based on self-reported information from patients in interviews.

In conclusion, the present study shows that DHAs such as ViViRA and Kaia can achieve significant improvements in pain and mobility independently of coaching. Coaching itself appears to strengthen health-related competence. Future studies should incorporate longer observation periods, objective usage data, and more comprehensive surveys of key variables in order to enable more differentiated conclusions.

## Supplementary Information

Below is the link to the electronic supplementary material.

Supplementary Material 1

## Data Availability

The data sets are available on reasonable request from the corresponding author.

## Abbreviations

AI: Artificial intelligence
ASAS: Assessment of SpondyloArthritis international Society
BASDAI: Bath Ankylosing Spondylitis Disease Activity Index
BASFI: Bath Ankylosing Spondylitis Functional Index
BASMI: Bath Ankylosing Spondylitis Metrology Index
BSA-Sport: Bewegungs- und Sportaktivität Fragebogen
BMI: Body mass index
CLBP: Chronic lower back pain
CI: Confidence interval
CE: Conformité Européenne
CG: Control Group
DHAs: Digital health applications
DRKS: Deutsches Register Klinischer Studien
EMM: Estimated marginal means
EULAR: European Alliance of Associations for Rheumatology
FITT: Frequency-Intensity-Time-Type
FACIT-F: Functional Assessment of Chronic Illness Therapy – Fatigue
FU: Follow-Up
HAQ: Health Assessment Questionnaire
IC: Intervention Coaching
IPAQ: International Physical Activity Score
MET: Metabolic Unit
PROMs: Patient reported Outcome Measurements
PAHCO: Physical Activity-related Health Competence
PA: Physical Activity
PSQI: Pittsburgh Sleep Quality Index
RedCap: Research Electronic Data Capture
QoL: Quality of life
SF-36: Short Form-Health-Survey-36
SpAs: Spondyloarthropathies
SD: Standard derivation
TSK: Tampa Scale of Kinesiophobia
VAS: Visual analog scale
