# Supplementary material for: Impact of personalized coaching on the use of digital health interventions for movement therapy in rheumatology: a randomized controlled trial
Source: Sci Rep. 2026 Jun 24;16:19582. doi: 10.1038/s41598-026-59770-7 (PMC13294491; doi:10.1038/s41598-026-59770-7)
Supplement: Supplementary file 1 — Supplementary Material 1 [file 41598_2026_59770_MOESM1_ESM.docx]

**Supplementary material**

**Supplement S1. Flow Diagram**

## Follow-Up 2

## Follow-Up 1

## Analysis

## Allocation

Analysed (n=27)
♦ Excluded from analysis (n=0)

Analysed (n=25)
♦ Excluded from analysis (n=0)

Analysed (n=26)
♦ Excluded from analysis (n=0)

Lost to follow-up 2 (n= 7; reasons: loss of interest n=5, medical reasons n= 2)

Lost to follow-up 2 (n= 6; reasons: loss of interest n=4, medical reasons n= 2)

Lost to follow-up 2 (n= 6; reasons: loss of interest n=6,)

Lost to follow-up 1 (n= 2; reasons: loss of interest n=1, no contact n= 1)

Lost to follow-up 1 (n= 5; reasons: loss of interest n=4, no contact n= 1)

Lost to follow-up 1 (n= 1; reasons: loss of interest n=1)

* Contact methods: phone, mail, poster; recruitment sources: Clinical trial outpatient unit; University medical outpatient department of Universitätsklinikum Erlangen-Nürnberg

Excluded (n=486)

♦  Not meeting inclusion criteria (n=59)

♦  Declined to participate (n=427)

♦  Other reasons (n=0)

Lost to follow-up 2 (give reasons) (n= 7)

Lost to follow-up 2 (give reasons) (n= 6)

Lost to follow-up 2 (give reasons) (n= 6)

Lost to follow-up 1 (give reasons) (n= 2)

Lost to follow-up 1 (give reasons) (n= 1)

Allocated to intervention 3 (IC Kaia) (n=27)

♦ Received allocated intervention (n=26)

♦ Did not receive allocated intervention (n=1; reason: pregnancy)

Allocated to intervention 2 (IC ViViRA) (n=25)

♦ Received allocated intervention (n=25)

♦ Did not receive allocated intervention (n=0)

Allocated to intervention 1 (CG ViViRA) (n=26)

♦ Received allocated intervention (n=26)

♦ Did not receive allocated intervention (n=0)

Randomized (n=78)

Assessed for eligibility (n=564)

## Enrollment

**Supplement S2. App-specific exclusions**

**Kaia**

Contraindications with ICD-10 code

- Possible spinal injuries (T09.- / M51.-)
- Reduced bone density (M80.- / M81.- )
- Spinal tumor (D48.- / C79.5)
- Spinal infections (M46.-)
- Herniated disc (M51.-)
- Previous spinal surgery (Z98.-)
- Advanced heart disease (I50.- / I51.-)
- Diseases that reduce control over the legs (G82.-)
- Diseases of the joints of the legs (M25.-)
- Pregnancy (O09.-)
- Unsteadiness (R26.-), frequent falls (R29.6)
- Tendency to bleed (D68.-), history of increased bleeding

Relative contraindications

- Kaia Back Pain should not be used if patients do not understand the instructions for use or do not have sufficient command of the language.
- Kaia Back Pain should not be used if patients are younger than 18 years of age.

The following additional exclusion criteria apply, unless a doctor has determined that the use of Kaia Back Pain is safe in the specific case of the patient:

- Cognitive impairments that do not allow the recommended use of Kaia Back Pain
- Physical impairments that do not allow the recommended use of Kaia Back Pain
- Age > 65 years
- Specific cause for the current episode of back pain (specific back pain)
- Non-specific chronic constant back pain

**ViViRA**

Contraindications with ICD-10 code

- G55.1 Compression of nerve roots and nerve plexus in disc disorders
- G99.2 Myelopathy in diseases classified elsewhere
- I80 Thrombosis, phlebitis, and thrombophlebitis
- M00 Purulent arthritis
- M01 Direct joint infections in infectious and parasitic diseases classified elsewhere
- M02 Reactive arthritis
- M03 Post-infectious and reactive arthritis in diseases classified elsewhere
- M23.4 Loose body in knee joint
- M24.05 Loose body: pelvic region and thigh [pelvis, femur, buttocks, hip, hip joint, sacroiliac joint]
- M50 Cervical disc disorders
- M50.1 Cervical disc disorder with radiculopathy
- M51.0 Lumbar and other disc disorders with myelopathy
- M51.1 Lumbar and other disc damage with radiculopathy
- M87 Bone necrosis
- M93.2 Osteochondrosis dissecans
- T84 Complications due to orthopedic endoprostheses, implants, or grafts

Absolute contraindications according to the instructions for use that are not coded according to ICD-10

- Acute inflammation or fever
- Spinal or joint infection

Relative exclusion criteria that can be coded according to ICD-10 and are at the discretion of the service provider.

- M46 Other inflammatory spondylopathies
- Z96.6 Presence of orthopedic joint implants
- Z96.64 Presence of a hip joint prosthesis
- Z96.65 Presence of a knee joint prosthesis
- Z96.68 Presence of other specified orthopedic joint implants

Relative exclusion criteria that cannot be coded according to ICD-10 and are at the discretion of the service provider.

- Undergoing neurological treatment
- Possible injuries to the spine, spinal cord, knee or hip joint
- Heart disease
- Rheumatic disease
- Reduced bone density
- Unsteady gait or frequent falls
- Tendency to bleed, history of increased bleeding or use of anticoagulant medication
- Pregnancy
- Cancer

**Supplement S3. Adherence and compliance Questionnaires**

**Compliance Questionnaire**

How often did you practice with the ViViRA or Kaia Health app?

1. Once a week
2. 2-3 times a week
3. 4-6 times a week
4. Once a day

How did you find the difficulty level of the exercises?

1. Very easy
2. Easy
3. Just right
4. Difficult
5. Very difficult

Did you miss having someone there to check your form and correct you if necessary?

1. Yes
2. No
3. Maybe
4. Don't know

Do you feel that using the app has had a positive effect on your mobility?

1. Yes
2. No
3. Maybe
4. Don't know

Do you feel that using the app has had a positive effect on your pain symptoms?

1. Yes
2. No
3. Maybe
4. Don't know

Would you continue to use the app outside of the study?

1. Yes
2. No
3. Maybe
4. Don't know

**Adherence Questionnaire**

Duration of rheumatic disease

1. < 1 year
2. 1-5 years
3. > 5 years

Which training method did you use?

1. Training with app
2. Training with guidance

How regularly did you do the training?

1. Always (100%)
2. Frequently (>75%)
3. Occasionally (< 50%)
4. Rarely (< 25%)

What was your main motivation for training? (Multiple answers possible)

1. Improvement of health
2. Relief of symptoms
3. Interest in the study
4. Recommendation by doctor/therapist

Other motivations: Free text response

Comprehensibility of the exercises

1. Very good
2. Good
3. Average
4. Poor

Support (if applicable)

1. Very good
2. Good
3. Average
4. Poor

Flexibility (e.g., training times)

1. Very good
2. Good
3. Average
4. Poor

What positive effects have you noticed? (e.g., less pain, more strength) Free text response

Were there any negative effects?

1. No
2. Yes

Which ones? Free text response

Were there any obstacles that made it difficult for you to participate?

1. Lack of time
2. Pain or flare-ups
3. Problems with the method (app or guided strength training)
4. Other

Did you need or want support?

1. No
2. Yes

What kind? Free text response

**Supplement S4. Protocol for IC ViViRA + personal coaching**

1. Organizational Matters & General Information

- Organizational notes and clarification of open questions
- Topic: Integrating physical activity into everyday life
- Goals:
  - Strengthen motivation and enjoyment of movement
  - Support sustainable implementation

2. Current Symptoms & Physical Activity Behavior

- Current well-being
- Weekly physical activity:
  - Frequency, duration, consistency
  - Fixed training days vs. spontaneous activity
- Enjoyment of movement:
  - What activities do you enjoy?
  - Do you notice positive effects from exercising?
    - Improvement or worsening of symptoms?
    - Onset of pain?

3. Use of the App

- Recommendation: Practice regularly (1–5 times per week), integrate realistically into daily routine
- Important notes:
  - Don’t set goals too high – start slowly
  - Avoid frustration due to overexertion
  - Supplement with physiotherapy (PT) if needed

4. Coaching Content

4.1 Health Education – Why Exercise?

Exercise supports:

- Physical performance
- Metabolism
- Maintenance of bone density, muscle mass & joint function
- Brain circulation & mood (antidepressant effect)

Exercise helps prevent:

- Progressive stiffening
- Pain
- Inflammation

4.2 Using the App & Exercise Routines

Key principles:

- Choose activities you enjoy
- Start with a comfortable intensity and duration
- Gradually increase:
  - Duration
  - Frequency
  - Intensity

During a flare-up or under stress:
→ Reduce intensity and volume!

4.3 Motivation: Reflection & Goal Setting

Reflection questions:

- What advantages and disadvantages do you see if you imagine exercising tonight or this weekend?
- What is your personal motivation?
  - Short-term: e.g., relaxation, improved mood
  - Long-term: e.g., disease progression, weight, comorbidities
- What obstacles are you facing? → Develop possible solutions

4.4 Motivation Strategies

a) Routines & Commitment:

- Schedule workouts in your calendar & check them off
- Use reminder functions
- Create a training plan
- Group workouts for additional support

b) Develop a Positive Perspective:

- Consciously recognize your own progress
- Reflect on emotional and health benefits

c) Curiosity & Variety:

- Try new types of physical activity
- Use the study/coaching period as a space to experiment
- Set realistic goals

d) Extrinsic Motivation:

- Reward systems (e.g., savings jar, activity tracker)

e) Reducing Fear:

- Fear reduces performance → promotes avoidance
- Recommendation: If struggling, take a step back
- Feel free to reach out with questions at any time

**Supplement S5. Baseline Characteristics**

| **Variable** | | **Baseline** | | | | **p-value***^2^* |
| --- | --- | --- | --- | --- | --- | --- |
|  |  | **Overall** | **CG ViViRA** | **IG ViViRA** | **IG Kaia** |  |
|  |  | N = 78*^1^* | N = 26*^1^* | N = 25*^1^* | N = 27*^1^* |  |
| Age (years) | | 51 (13) | 52 (15) | 51 (12) | 51 (11) | 0.8 |
| Sex | Total |  |  |  |  | 0.088 |
|  | Female | 51 (68%) | 16 (64%) | 21 (84%) | 14 (56%) |  |
| Body mass index (kg/m2) | | 26.8 (5.5) | 28.1 (6.8) | 25.7 (4.6) | 26.5 (4.7) | 0.3 |
| Disease duration (months) | | 102 (71) | 94 (70) | 81 (55) | 132 (81) | 0.067 |
| BASDAI | | 4.30 (1.91) | 4.27 (2.19) | 4.22 (1.91) | 4.39 (1.67) | 0.9 |
| BASFI | | 1.87 (1.80) | 2.05 (2.02) | 1.43 (1.48) | 2.11 (1.84) | 0.3 |
| BASMI | BASMI total | 1.98 (1.15) | 1.96 (1.14) | 1.94 (1.09) | 2.05 (1.26) | >0.9 |
|  | Lumbar flexion | 8.9 (3.6) | 9.8 (4.6) | 9.1 (2.8) | 8.0 (3.1) | 0.3 |
|  | Spinal lateral flexion | 14.5 (4.5) | 14.5 (5.1) | 13.6 (3.2) | 15.2 (4.9) | 0.4 |
|  | Tragus wall distance | 11.26 (2.44) | 11.03 (2.00) | 11.36 (2.56) | 11.40 (2.78) | >0.9 |
|  | Maximum intermalleolar distance | 107 (22) | 108 (20) | 105 (24) | 108 (23) | 0.9 |
|  | Cervical rotation | 66 (14) | 67 (11) | 69 (12) | 63 (17) | 0.4 |
|  | Modified Schober test | 0.95 (1.60) | 1.00 (1.92) | 0.56 (1.00) | 1.26 (1.70) | 0.3 |
| HAQ result | | 0.54 (0.47) | 0.50 (0.46) | 0.48 (0.50) | 0.64 (0.45) | 0.4 |
| Handgrip max right | | 76 (30) | 78 (31) | 67 (26) | 81 (32) | 0.3 |
| Handgrip max left | | 69 (30) | 70 (31) | 63 (27) | 75 (31) | 0.4 |
| IPAQ total MET | | 6,902 (14,756) | 5,705 (9,179) | 10,554 (23,381) | 4,445 (4,330) | 0.2 |
| IPAQ sitting min per week | | 1,732 (956) | 1,880 (1,104) | 1,675 (756) | 1,640 (999) | 0.8 |
| TSK score | | 35 (8) | 35 (7) | 32 (8) | 37 (8) | 0.052 |
| PAHCO | movement sum | 10.4 (3.6) | 11.1 (3.9) | 9.9 (3.4) | 10.1 (3.5) | 0.4 |
|  | movement mean | 2.36 (0.83) | 2.52 (0.90) | 2.25 (0.78) | 2.29 (0.80) | 0.4 |
|  | control sum | 5.96 (2.35) | 6.25 (2.29) | 5.53 (2.40) | 6.10 (2.41) | 0.5 |
|  | control mean | 2.21 (0.87) | 2.32 (0.85) | 2.05 (0.89) | 2.26 (0.89) | 0.5 |
|  | self-regulation sum | 9.88 (2.38) | 9.84 (3.00) | 10.03 (1.92) | 9.78 (2.13) | 0.8 |
|  | self-regulation mean | 2.67 (0.64) | 2.66 (0.81) | 2.71 (0.52) | 2.64 (0.57) | 0.8 |
| Patient VAS activity | | 4 (2) | 5 (2) | 4 (2) | 5 (2) | 0.6 |
| GAP total score | | 5 (2) | 4 (2) | 4 (2) | 5 (2) | 0.10 |
| GAP pain | | 5 (2) | 4 (3) | 5 (2) | 5 (2) | 0.4 |
| Patient VAS pain | | 4 (2) | 4 (3) | 4 (2) | 5 (2) | 0.7 |
| Patient VAS back pain | | 5 (3) | 4 (3) | 4 (2) | 5 (3) | 0.4 |
| Pain total | | 15.7 (6.4) | 15.4 (6.3) | 16.4 (5.4) | 15.2 (7.5) | 0.8 |
| Pain sum | | 16.9 (6.7) | 16.6 (6.3) | 17.7 (5.7) | 16.4 (8.0) | 0.8 |
| KSK score | | 39 (11) | 39 (12) | 41 (11) | 36 (9) | 0.2 |
| PSK score | | 43 (10) | 46 (10) | 44 (11) | 39 (9) | 0.081 |
| PSQI total score | | 13 (3) | 13 (2) | 13 (3) | 14 (4) | 0.3 |
| FACIT fatigue sum score | | 21 (10) | 20 (9) | 20 (12) | 24 (10) | 0.2 |
| Medication | csDMARDs | 22 (34%) | 5 (25%) | 9 (39%) | 8 (38%) | 0.6 |
|  | tsDMARDs | 11 (20%) | 6 (32%) | 2 (11%) | 3 (18%) | 0.3 |
|  | bDMARDs | 19 (32%) | 4 (21%) | 7 (37%) | 8 (36%) | 0.5 |
|  | Glucocorticoids | 6 (10%) | 3 (15%) | 1 (5.6%) | 2 (10%) | 0.9 |
|  | Additional meds | 42 (61%) | 13 (62%) | 12 (48%) | 17 (74%) | 0.2 |
| Physically active? | | 57 (78%) | 20 (80%) | 20 (83%) | 17 (71%) | 0.6 |
| PA amount | total |  |  |  |  | 0.6 |
|  | Low < 5×30 min/week | 25 (45%) | 7 (35%) | 9 (47%) | 9 (56%) |  |
|  | Moderate 5×30 min/week | 21 (38%) | 8 (40%) | 7 (37%) | 6 (38%) |  |
|  | High > 5×30 min/week | 9 (16%) | 5 (25%) | 3 (16%) | 1 (6.3%) |  |
| Physiotherapy | ever | 60 (80%) | 17 (68%) | 21 (84%) | 22 (88%) | 0.2 |
|  | currently | 35 (58%) | 11 (65%) | 11 (52%) | 13 (59%) | 0.7 |
| *^1^* Mean (SD); n (%) | | | | | | |
| *^2^* Kruskal-Wallis rank sum test; Fisher’s exact test; Pearson’s Chi-squared test | | | | | | |

**Supplement S6. Observed outcome values by intervention group and visit**

| **Outcome** | **Arm** | **Visit** | **Mean (SD)** | **N** |
| --- | --- | --- | --- | --- |
| BASDAI | Arm 1: ViViRA | Baseline Screening | 4.3 (2.2) | 25 |
| BASDAI | Arm 2: ViViRA + Coaching | Baseline Screening | 4.2 (1.9) | 24 |
| BASDAI | Arm 3: Kaia + Coaching | Baseline Screening | 4.4 (1.7) | 25 |
| BASDAI | Arm 1: ViViRA | Follow-Up-1 (3 Months) | 3.9 (2.0) | 21 |
| BASDAI | Arm 2: ViViRA + Coaching | Follow-Up-1 (3 Months) | 4.1 (2.3) | 24 |
| BASDAI | Arm 3: Kaia + Coaching | Follow-Up-1 (3 Months) | 4.2 (2.1) | 24 |
| BASDAI | Arm 1: ViViRA | Follow-Up-2 (6 Months) | 3.5 (2.1) | 15 |
| BASDAI | Arm 2: ViViRA + Coaching | Follow-Up-2 (6 Months) | 3.8 (2.2) | 18 |
| BASDAI | Arm 3: Kaia + Coaching | Follow-Up-2 (6 Months) | 4.1 (1.7) | 18 |
| BASFI | Arm 1: ViViRA | Baseline Screening | 2.0 (2.0) | 25 |
| BASFI | Arm 2: ViViRA + Coaching | Baseline Screening | 1.4 (1.5) | 24 |
| BASFI | Arm 3: Kaia + Coaching | Baseline Screening | 2.1 (1.8) | 25 |
| BASFI | Arm 1: ViViRA | Follow-Up-1 (3 Months) | 2.0 (2.0) | 21 |
| BASFI | Arm 2: ViViRA + Coaching | Follow-Up-1 (3 Months) | 1.7 (1.8) | 24 |
| BASFI | Arm 3: Kaia + Coaching | Follow-Up-1 (3 Months) | 2.2 (1.9) | 24 |
| BASFI | Arm 1: ViViRA | Follow-Up-2 (6 Months) | 2.1 (2.1) | 15 |
| BASFI | Arm 2: ViViRA + Coaching | Follow-Up-2 (6 Months) | 1.6 (1.6) | 18 |
| BASFI | Arm 3: Kaia + Coaching | Follow-Up-2 (6 Months) | 2.2 (1.7) | 18 |
| BASMI total | Arm 1: ViViRA | Baseline Screening | 2.0 (1.1) | 24 |
| BASMI total | Arm 2: ViViRA + Coaching | Baseline Screening | 1.9 (1.1) | 25 |
| BASMI total | Arm 3: Kaia + Coaching | Baseline Screening | 2.1 (1.3) | 27 |
| BASMI total | Arm 1: ViViRA | Follow-Up-1 (3 Months) | 1.2 (1.0) | 17 |
| BASMI total | Arm 2: ViViRA + Coaching | Follow-Up-1 (3 Months) | 1.3 (0.8) | 19 |
| BASMI total | Arm 3: Kaia + Coaching | Follow-Up-1 (3 Months) | 1.5 (0.9) | 22 |
| FACIT fatigue sum score | Arm 1: ViViRA | Baseline Screening | 20.2 (9.1) | 25 |
| FACIT fatigue sum score | Arm 2: ViViRA + Coaching | Baseline Screening | 19.8 (11.7) | 24 |
| FACIT fatigue sum score | Arm 3: Kaia + Coaching | Baseline Screening | 24.3 (10.1) | 25 |
| FACIT fatigue sum score | Arm 1: ViViRA | Follow-Up-1 (3 Months) | 16.6 (8.7) | 21 |
| FACIT fatigue sum score | Arm 2: ViViRA + Coaching | Follow-Up-1 (3 Months) | 17.8 (13.1) | 23 |
| FACIT fatigue sum score | Arm 3: Kaia + Coaching | Follow-Up-1 (3 Months) | 20.6 (8.9) | 24 |
| FACIT fatigue sum score | Arm 1: ViViRA | Follow-Up-2 (6 Months) | 16.5 (11.5) | 15 |
| FACIT fatigue sum score | Arm 2: ViViRA + Coaching | Follow-Up-2 (6 Months) | 16.7 (13.6) | 18 |
| FACIT fatigue sum score | Arm 3: Kaia + Coaching | Follow-Up-2 (6 Months) | 21.1 (9.0) | 18 |
| PAHCO control sum | Arm 1: ViViRA | Baseline Screening | 6.3 (2.3) | 25 |
| PAHCO control sum | Arm 2: ViViRA + Coaching | Baseline Screening | 5.5 (2.4) | 24 |
| PAHCO control sum | Arm 3: Kaia + Coaching | Baseline Screening | 6.1 (2.4) | 24 |
| PAHCO control sum | Arm 1: ViViRA | Follow-Up-1 (3 Months) | 6.8 (1.7) | 21 |
| PAHCO control sum | Arm 2: ViViRA + Coaching | Follow-Up-1 (3 Months) | 6.4 (2.4) | 23 |
| PAHCO control sum | Arm 3: Kaia + Coaching | Follow-Up-1 (3 Months) | 6.5 (2.3) | 24 |
| PAHCO control sum | Arm 1: ViViRA | Follow-Up-2 (6 Months) | 6.2 (1.6) | 15 |
| PAHCO control sum | Arm 2: ViViRA + Coaching | Follow-Up-2 (6 Months) | 6.6 (2.1) | 17 |
| PAHCO control sum | Arm 3: Kaia + Coaching | Follow-Up-2 (6 Months) | 7.0 (2.3) | 18 |
| PAHCO movement sum | Arm 1: ViViRA | Baseline Screening | 11.1 (3.9) | 25 |
| PAHCO movement sum | Arm 2: ViViRA + Coaching | Baseline Screening | 9.9 (3.4) | 24 |
| PAHCO movement sum | Arm 3: Kaia + Coaching | Baseline Screening | 10.1 (3.5) | 24 |
| PAHCO movement sum | Arm 1: ViViRA | Follow-Up-1 (3 Months) | 10.8 (3.5) | 21 |
| PAHCO movement sum | Arm 2: ViViRA + Coaching | Follow-Up-1 (3 Months) | 10.5 (3.6) | 23 |
| PAHCO movement sum | Arm 3: Kaia + Coaching | Follow-Up-1 (3 Months) | 9.8 (3.7) | 24 |
| PAHCO movement sum | Arm 1: ViViRA | Follow-Up-2 (6 Months) | 10.3 (4.4) | 15 |
| PAHCO movement sum | Arm 2: ViViRA + Coaching | Follow-Up-2 (6 Months) | 10.9 (3.7) | 17 |
| PAHCO movement sum | Arm 3: Kaia + Coaching | Follow-Up-2 (6 Months) | 9.6 (3.7) | 18 |
| PAHCO self-regulation sum | Arm 1: ViViRA | Baseline Screening | 9.8 (3.0) | 25 |
| PAHCO self-regulation sum | Arm 2: ViViRA + Coaching | Baseline Screening | 10.0 (1.9) | 24 |
| PAHCO self-regulation sum | Arm 3: Kaia + Coaching | Baseline Screening | 9.8 (2.1) | 24 |
| PAHCO self-regulation sum | Arm 1: ViViRA | Follow-Up-1 (3 Months) | 10.4 (2.2) | 21 |
| PAHCO self-regulation sum | Arm 2: ViViRA + Coaching | Follow-Up-1 (3 Months) | 10.0 (2.4) | 23 |
| PAHCO self-regulation sum | Arm 3: Kaia + Coaching | Follow-Up-1 (3 Months) | 9.9 (2.2) | 24 |
| PAHCO self-regulation sum | Arm 1: ViViRA | Follow-Up-2 (6 Months) | 10.3 (2.1) | 15 |
| PAHCO self-regulation sum | Arm 2: ViViRA + Coaching | Follow-Up-2 (6 Months) | 10.3 (2.6) | 17 |
| PAHCO self-regulation sum | Arm 3: Kaia + Coaching | Follow-Up-2 (6 Months) | 10.4 (1.9) | 18 |
| PSQI total score | Arm 1: ViViRA | Baseline Screening | 13.4 (2.1) | 25 |
| PSQI total score | Arm 2: ViViRA + Coaching | Baseline Screening | 12.8 (2.8) | 25 |
| PSQI total score | Arm 3: Kaia + Coaching | Baseline Screening | 14.0 (3.6) | 25 |
| PSQI total score | Arm 1: ViViRA | Follow-Up-1 (3 Months) | 12.2 (2.3) | 21 |
| PSQI total score | Arm 2: ViViRA + Coaching | Follow-Up-1 (3 Months) | 12.7 (3.7) | 24 |
| PSQI total score | Arm 3: Kaia + Coaching | Follow-Up-1 (3 Months) | 13.5 (2.4) | 23 |
| PSQI total score | Arm 1: ViViRA | Follow-Up-2 (6 Months) | 13.0 (2.1) | 15 |
| PSQI total score | Arm 2: ViViRA + Coaching | Follow-Up-2 (6 Months) | 12.4 (3.5) | 18 |
| PSQI total score | Arm 3: Kaia + Coaching | Follow-Up-2 (6 Months) | 13.2 (2.3) | 18 |
| Pain total | Arm 1: ViViRA | Baseline Screening | 15.4 (6.3) | 26 |
| Pain total | Arm 2: ViViRA + Coaching | Baseline Screening | 16.4 (5.4) | 25 |
| Pain total | Arm 3: Kaia + Coaching | Baseline Screening | 15.2 (7.5) | 27 |
| Pain total | Arm 1: ViViRA | Follow-Up-1 (3 Months) | 12.2 (7.9) | 26 |
| Pain total | Arm 2: ViViRA + Coaching | Follow-Up-1 (3 Months) | 14.4 (6.2) | 25 |
| Pain total | Arm 3: Kaia + Coaching | Follow-Up-1 (3 Months) | 15.2 (6.8) | 27 |
| Pain total | Arm 1: ViViRA | Follow-Up-2 (6 Months) | 8.8 (9.4) | 26 |
| Pain total | Arm 2: ViViRA + Coaching | Follow-Up-2 (6 Months) | 10.9 (8.9) | 25 |
| Pain total | Arm 3: Kaia + Coaching | Follow-Up-2 (6 Months) | 10.6 (9.5) | 27 |
| SF-36 MCS | Arm 1: ViViRA | Baseline Screening | 45.5 (10.5) | 25 |
| SF-36 MCS | Arm 2: ViViRA + Coaching | Baseline Screening | 44.1 (10.6) | 25 |
| SF-36 MCS | Arm 3: Kaia + Coaching | Baseline Screening | 39.2 (9.3) | 25 |
| SF-36 MCS | Arm 1: ViViRA | Follow-Up-1 (3 Months) | 46.4 (8.3) | 21 |
| SF-36 MCS | Arm 2: ViViRA + Coaching | Follow-Up-1 (3 Months) | 45.3 (11.5) | 24 |
| SF-36 MCS | Arm 3: Kaia + Coaching | Follow-Up-1 (3 Months) | 41.5 (8.5) | 25 |
| SF-36 MCS | Arm 1: ViViRA | Follow-Up-2 (6 Months) | 48.1 (9.4) | 15 |
| SF-36 MCS | Arm 2: ViViRA + Coaching | Follow-Up-2 (6 Months) | 46.9 (11.1) | 18 |
| SF-36 MCS | Arm 3: Kaia + Coaching | Follow-Up-2 (6 Months) | 44.7 (9.3) | 18 |
| SF-36 PCS | Arm 1: ViViRA | Baseline Screening | 39.5 (11.6) | 25 |
| SF-36 PCS | Arm 2: ViViRA + Coaching | Baseline Screening | 40.6 (10.6) | 25 |
| SF-36 PCS | Arm 3: Kaia + Coaching | Baseline Screening | 36.2 (9.1) | 25 |
| SF-36 PCS | Arm 1: ViViRA | Follow-Up-1 (3 Months) | 41.1 (11.1) | 21 |
| SF-36 PCS | Arm 2: ViViRA + Coaching | Follow-Up-1 (3 Months) | 40.6 (10.9) | 24 |
| SF-36 PCS | Arm 3: Kaia + Coaching | Follow-Up-1 (3 Months) | 38.0 (9.1) | 25 |
| SF-36 PCS | Arm 1: ViViRA | Follow-Up-2 (6 Months) | 41.2 (9.9) | 15 |
| SF-36 PCS | Arm 2: ViViRA + Coaching | Follow-Up-2 (6 Months) | 42.2 (10.7) | 18 |
| SF-36 PCS | Arm 3: Kaia + Coaching | Follow-Up-2 (6 Months) | 39.3 (9.7) | 18 |

Values are presented as mean (SD). N denotes the number of participants with available data at each assessment.

**Supplement S7. Self-reported training adherence**
